# Tailoring Nano-Porous Surface of Aligned Electrospun Poly (L-Lactic Acid) Fibers for Nerve Tissue Engineering

**DOI:** 10.3390/ijms22073536

**Published:** 2021-03-29

**Authors:** Hongyun Xuan, Biyun Li, Feng Xiong, Shuyuan Wu, Zhuojun Zhang, Yumin Yang, Huihua Yuan

**Affiliations:** 1 School of Life Sciences, Nantong University, Nantong 226019, China; hyxuan_seu@163.com (H.X.); libiyun1986@163.com (B.L.); xiongfengxl@163.com (F.X.); wsy0326_2021@163.com (S.W.); swgczzj@163.com (Z.Z.); 2 Key Laboratory of Neuroregeneration of Jiangsu and Ministry of Education, Co-Innovation Center of Neuroregeneration, Nantong University, Nantong 226001, China

**Keywords:** well-aligned nano-porous fibers, bacterial growth inhibition, cellular responses, nerve regeneration

## Abstract

Despite the existence of many attempts at nerve tissue engineering, there is no ideal strategy to date for effectively treating defective peripheral nerve tissue. In the present study, well-aligned poly (L-lactic acid) (PLLA) nanofibers with varied nano-porous surface structures were designed within different ambient humidity levels using the stable jet electrospinning (SJES) technique. Nanofibers have the capacity to inhibit bacterial adhesion, especially with respect to *Staphylococcus aureus* (*S. aureus*). It was noteworthy to find that the large nano-porous fibers were less detrimentally affected by *S. aureus* than smaller fibers. Large nano-pores furthermore proved more conducive to the proliferation and differentiation of neural stem cells (NSCs), while small nano-pores were more beneficial to NSC migration. Thus, this study concluded that well-aligned fibers with varied nano-porous surface structures could reduce bacterial colonization and enhance cellular responses, which could be used as promising material in tissue engineering, especially for neuro-regeneration.

## 1. Introduction

Peripheral and central nerve tissue defects have been the focus of constant attention for decades, and despite extensive clinical research endeavors, successful neuron repair still faces vast challenges due to various factors such as axonal outgrowth inhibition, neuron cell division restriction, and astrocyte dysfunction [1,2,3]. Nerve autografts are currently the preferred and optimal method for treating long nerve gap defects, but this technique is restricted due to the shortage of donor sites, risks of complications, the formation of neuroma, and lengthy and multiple surgical procedures [4,5,6]. With the development of tissue engineering techniques, especially neural tissue engineering, more bespoke treatments are called for in order to overcome the numerous medical obstacles mentioned above. Offering the advantages of high porosity, large superficial areas, and a tailorable surface, 3D porous nanofiber scaffolds offer the capacity to be functionally modified by biological molecules, carbohydrates, chemical compounds, and proteins [7,8]. Nanofibrous scaffolds that are supportive of cell adhesion, migration, proliferation, and differentiation, share similarities with the structure and function of natural extracellular matrix (ECM) [9,10,11,12]. Thus, biomimetic nanofibrous scaffolds are likely candidates to be selected for the purposes of performing autografts to repair neuronal defects. For instance, Kim et al. produced a porous and aligned polycaprolactone (PCL)/silk/quercetin fibrous scaffold to enhance neural cell adhesion, migration, and direction of growth, which resulted in increased neural regeneration [13]. Evidently, alignment of electrospun fibers for nanotopographical guidance of nerve cells is critical for nerve tissue engineering.

Electrospun fiber surface nanotopography has been known to regulate cell function for various tissue engineering applications [14,15,16,17,18,19,20,21]. This nanotopography improves human mesenchymal stem cell proliferation and migration [14], reduces RAW 264.7 cell elongation [15], modestly regulates cytokine expression of macrophages [16], and modulates skeletal differentiation of human mesenchymal stromal cells [17]. We previously demonstrated that aligned electrospun poly (L-lactic acid) (PLLA) fibers with elliptical nano-pore surfaces enhanced the cellular response of vascular smooth muscle cells [18]. Special for never tissue engineering, Zamani et al. showed that electrospun poly (lactic-co-glycolic acid (PLGA) porous cylindrical fibers with nanometer surface roughness improved the attachment, growth, and proliferation of human A-172 nerve cells [19]. Yang et al. reported that aligned electrospun PLLA pore fibers increased the alignment degree of neurite outgrowth and neurite length of pheochromocytoma (PC12) cells [20]. Johnson et al. revealed that electrospun fiber surface nanotopography could influence astrocyte elongation and the capability of astrocytes to direct neurites [21]. Although aligned electrospun fiber scaffold have been extensively investigated in never tissue engineering, little is known about the influence of introducing nanotopography on their surface for neural stem cell (NSC) differentiation.

Bacterial contamination of medical implants is associated with bacteria biofilm formation and resulting in implant rejection, which remains a critical problem in tissue engineering [22,23,24]. Antibiotics treatment was often used to kill the bacteria on surgical implants. However, due to antibiotic abuse, bacteria has developed into antibiotic-resistant bacteria that would lead to more deaths than cancer in future decades [25]. Reduced microorganism colonization with an implant might be another effective way to enable the immune system to eliminate bacteria, which was found to be capable of resolving the issue of bacterial contamination of surgical implants [26,27]. Previous studies have reported that micro/nano-topographies could prevent implant surfaces from microorganism colonization [28,29,30,31]. Recently, Machado-Paula et al. initially generated macro-fibers with special topography surfaces by rotary jet spinning in order to protect bacterial colonization [32]. In this sense, engineering electrospun aligned nanofiber surfaces with nano-topographies could be taken into consideration when designing electrospun biomimetic fibrous scaffolds for never tissue engineering. However, little knowledge on the influence of electrospun aligned nanofiber surface nano-topographies on bacterial colonization is available.

Herein, we aimed to provide the first report to investigate the influence of electrospun aligned nanofiber surface nano-topographies on bacterial colonization and the differentiation of NSCs. Well-aligned nano-porous PLLA nanofibers were generated under various ranges of ambient humidity on the basis of our previous work [18]. Fiber surface roughness, morphology, crystallinity, and wettability were investigated using various characterization techniques. Moreover, the nano-porous PLLA fibers were cultured with bacteria to explore the effects of fiber nano-pore surfaces on bacterial colonization. In addition, the fibers were seeded with NSCs to observe the influence of nano-porous fibers on cell adhesion, migration, proliferation, and differentiation.

## 2. Results and Discussion

### 2.1. Preparation and Characterization of Aligned Electrospun PLLA Pore Fibers

**Figure 1:**
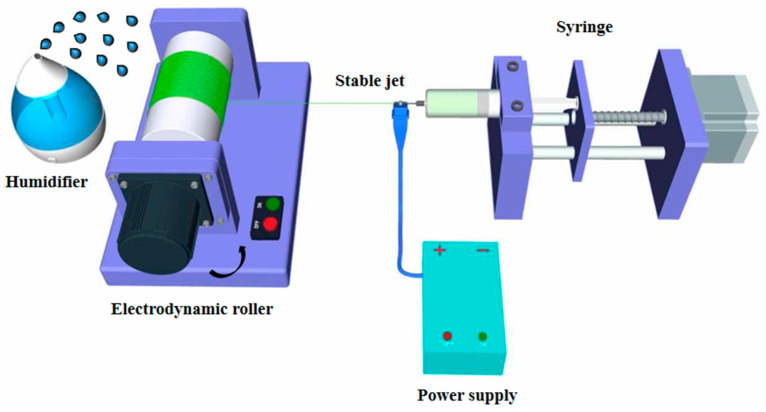
Schematic diagram of fabrication of the aligned nano-porous poly (L-lactic acid) (PLLA) fibers under various humidity environments via the stable jet electrospinning (SJES) method.

**Figure 2:**
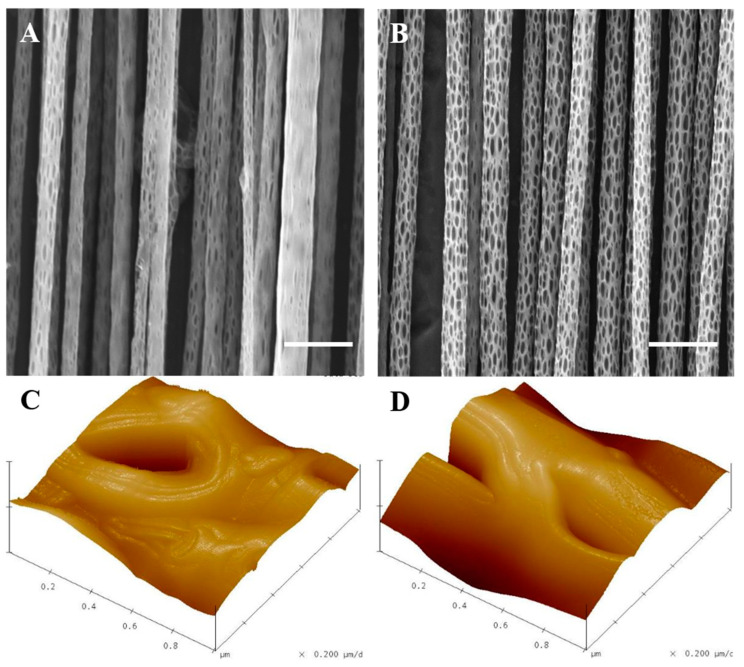
SEM (**A**,**B**) and atomic force microscopy (AFM) (**C**,**D**) images of the aligned nano-porous PLLA fibers obtained via stable jet electrospinning at ambient humidity of 40% (RH40) and 70% (RH70). Scale bar = 5 μm.

The SJES method had been developed to produce well-aligned nano-porous PLLA fibers under various ambient humidity conditions (40% and 70%). This was achieved by forming a fairly extended, constant, linear jet measuring several tens of centimeters in length (Figure 1). SEM and AFM images revealed the presence of a large number of nano-scaled ellipsoidal pores with their major axis along the direction of the fiber on the surfaces of the well-aligned single PLLA ultrafine fiber (Figure 2). Fiber surfaces were filled with more and larger ellipsoidal nano-pores as humidity levels increased. Specifically, the mean diameter of ellipsoidal nano-pores fibers measured 1.7 ± 0.2 μm at 70% humidity (RH70), which was slightly higher than the 1.4 ± 0.3 μm determined at 40% humidity (RH40) (Figure 2A,B). However, there was no significant differences between two different humidities, indicating that varying humidity had negligible effect on the resultant fiber finesse. Pore structure on electrospun ultrafine fibers could be achieved through various approaches including varying collector temperature, solvent and ambient humidity, nanoimprint lithography, wet chemical etching methods, and gas plasma [15,33,34]. In the current study, the solvent and relative humidity method was chosen because it is a simple and direct process without any post treatment, higher flexibility in controlling the roughness, and homogeneous treatment of each single fiber [17,18]. Indeed, the results above have clearly showed that the nano-pore on electrospun aligned PLLA ultrafine fiber surfaces could be easily controlled by relative humidity and enlarged with increased humidity. The mechanism of nano-pore formation on aligned PLLA ultrafine fibers is correlated with the phase separation (induced by vapor, thermal, or non-solvent) and breath figure effect [17,18]. Moreover, ellipsoidal-like nano-pores on the aligned PLLA fiber surfaces were oriented in the direction of the long axis. This was due to the fact that the electric field jet, which extended in a uniaxial direction, also provided torque from the rotary drum in the process of electrospinning [18].

AFM was also employed to explore the effects of ambient humidity variations on the topology of nano-pores. The results showed that RH40 and RH70 fibers presented different surface roughnesses, namely, 66 ± 10 nm and 122 ± 27 nm (Figure 2C,D), respectively. It was suggested that the ambient humidity during the SJES process could alter nano-porous surface texture when fiber surfaces presented variable roughness, which was consistent with the SEM data mentioned above. This is in good compliance with previous studies as well [17,18].

**Figure 3:**
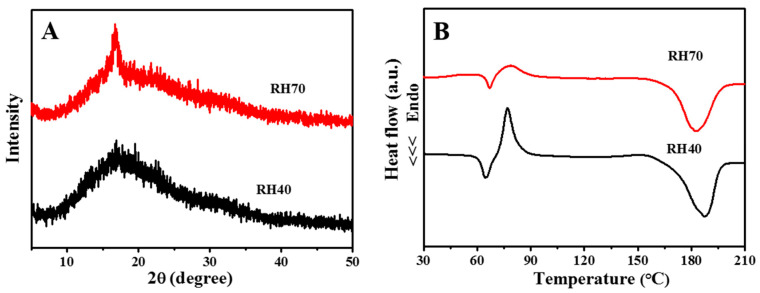
XRD patterns (**A**) and differential scanning calorimetry (DSC) thermograms (**B**) of the aligned nano-porous PLLA fibers.

XRD was used to investigate the crystallinity of the RH40 and RH70 fibers. It is noteworthy that at around 2ϑ = 16°, a peak appeared in all XRD patterns of the PLLA fibers obtained under different ambient humidity conditions, which corresponded to α-form crystals [35]. The greater intensity in the RH70 nano-porous fibers denoted the higher crystallinity of electrospun PLLA fibers with RH70 (Figure 3A). Figure 3B shows the DSC thermograms of RH40 and RH70 nano-porous fibers. The glass transition temperatures (*T_g_*) for RH40 and RH70 were about 62 and 65 °C, respectively. This was ascribed to the fact that the thermal vibration of the PLLA molecular segment was restrained after the increase in nano-pore size on the PLLA fiber surfaces. The cold crystallization peak (*T_c_*) of RH40 and RH70 increased from 77 °C to 79 °C due to the large nano-pores restricting the formation of hydrogen bonds between PLLA molecules. The melting temperatures (*T_m_*) were approximately 180 °C, indicating that the nano-pores had no effect on the formation of α-form PLLA crystals [35]. The crystallinities (Xc%, calculated by Equation (1)) of RH40 and RH70 PLLA fibers were 36% and 45%, respectively. The crystallinity of RH70 nano-pore PLLA fibers noticeably increased, which was consistent with the XRD results. Similar results were also reported by previous studies [35]. These findings are very important because crystallinity affects polymer physical configuration, which shows important characteristics that control bacterial adhesion and cellular responses [18,32].

**Figure 4:**
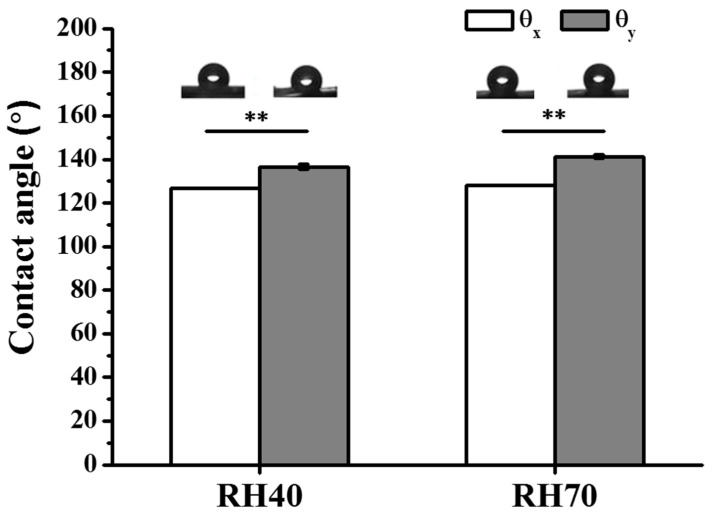
Water contact angle photographs of the aligned nano-porous PLLA fibers (** *p* < 0.01).

It is known that surface roughness can affect scaffold wettability and thus affects bacterial and cellular responses [18,32]. Therefore, surface wettability of a scaffold allows one to predict the hydrophilicity/hydrophobicity of the scaffold and consequently bacterial and cellular susceptibility. The wettability of nano-porous aligned PLLA fibers was evaluated by WCA (Figure 4). Figure 4 shows that both WCAs in the *x*-direction (*ϑ_x_*) were smaller than in the *y*-direction (*ϑ_y_*) with a featureless surface of anisotropic wettability, which suggests a faster spreading speed for water droplets along the fiber axis than in the perpendicular direction because of the existence of energy barrier to wetting [36,37]. Moreover, the increasing size of nano-pores, when the *x*/*y* direction contact angle indices (|*ϑ_x_*−*ϑ_y_*|) were augmented from 9.96 (RH40) to 13.1 (RH70) (Figure 4), which demonstrated that the air stably fixed in the electrospun fiber pore surface resulted in the increased hydrophobicity [16]. Together, these results illustrated that the nano-porous surface of individual fibers demonstrated a sound capacity to adjust the aligned PLLA fibers’ wettability. Increasing the surface roughness or nano-pore size of these fibers furthermore contributed to enhancing surface hydrophobicity.

### 2.2. Bacterial Activity in Response to Aligned Electrospun PLLA Pore Fibers

**Figure 5:**
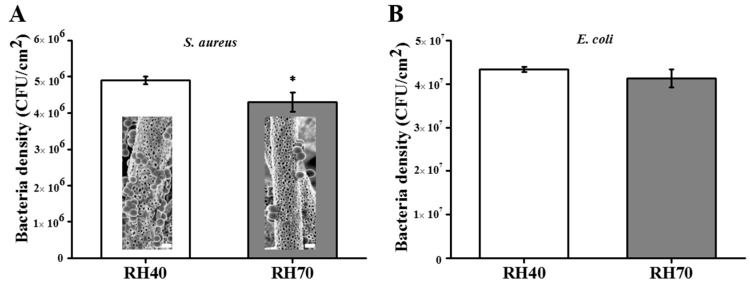
Colony-forming units of *Staphylococcus aureus* (*S. aureus*) (**A**) and *Escherichia coli* (*E. coli*) (**B**). Inset image in (**A**) is SEM images of *S. aureus* on the RH40 and RH70 fibers, Scale bar is 1 μm (* *p* < 0.05).

Bacteria colonization on the scaffolds was determined here through CFUs and SEM. Figure 5A shows the CFUs for *S. aureus* (Gram-positive) and Figure 5B for *E. coli* (Gram-negative). Clearly, the RH70 fibers showed reduced colonization from both bacteria strains as compared to the RH40 fibers. Interestingly, the RH70 fibers showed a significant difference in the reduction of *S. aureus* colonization and no difference for *E. coli*. The difference between Gram-positive and Gram–negative bacteria may correlate with the bacterial wall structure. The Gram-positive bacteria presented a simple surface made up of a membrane and a few layers of peptidoglycans. In contrast, the surface of the Gram-negative bacteria was complex, consisting of one membrane, some layers of peptidoglycans, and one external membrane comprising a capsule of polysaccharides and lipopolysaccharides (LPS). Gram-negative bacteria presented much greater resistance to surface geometries because of the surface complexity. Furthermore, evidence showed that the presence of LPS in the membrane played a critical role in incipient bacterial adhesion to surfaces. Moreover, the capsule consisting of primarily long-chain polysaccharides provided increasing resistance as an external barrier to bacterial protection [38,39]. The Gram-negative bacteria displayed better bacterial colonization, implying that surface morphology may have been overcome by the bacterial wall structure. The inset SEM image in Figure 5A also displays much more adherent *S. aureus* covering the RH40 fibers than the RH70 fibers. These results suggested that the larger nano-pore RH70 fiber could inhibit *S. aureus* colonization on the surface and may facilitate its disinfection. However, nano-pore RH70 fibers in this study only showed a physical interference, but they did not kill bacteria. In the future, the nano-pore fibrous scaffolds with the addition of some antibiotics will extend their biological applications.

### 2.3. NSC Differentiation on Aligned Electrospun PLLA Pore Fibers

**Figure 6:**
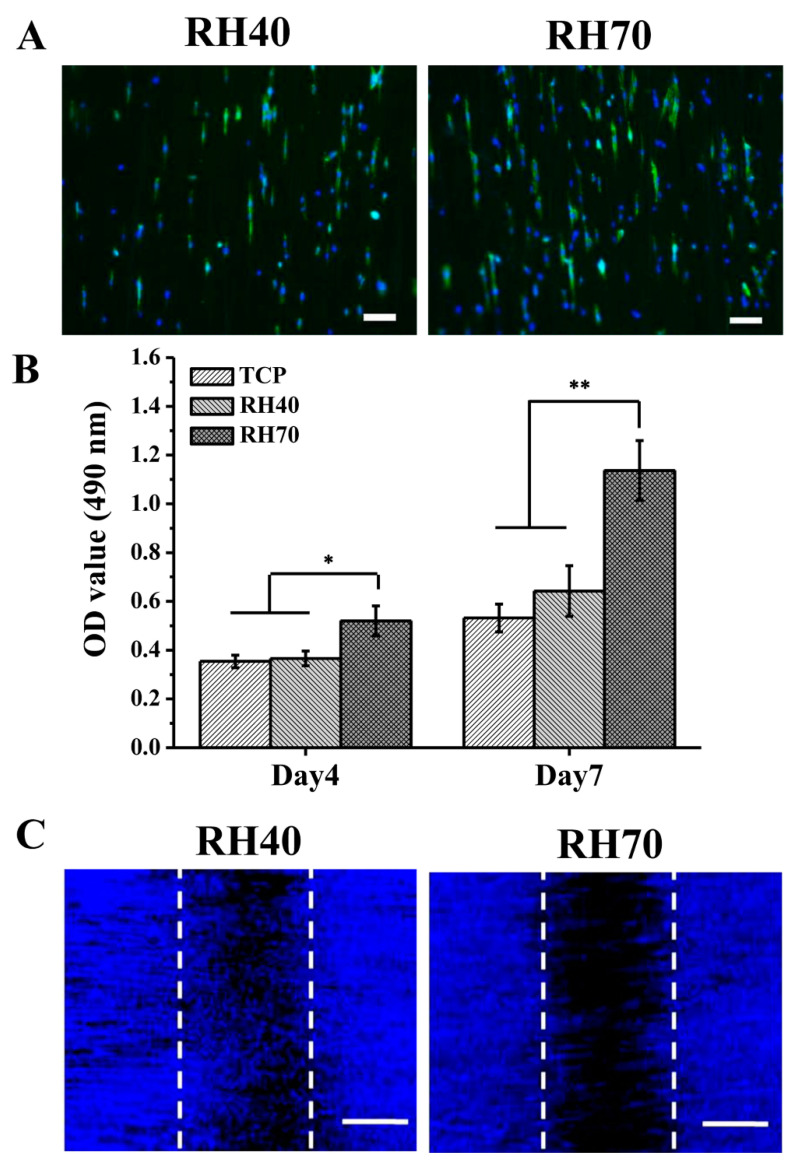
(**A**) Immunofluorescence images of neural stem cells (NSCs) cultured on RH40 and RH70 for 1 day. The green staining is actin, and the blue staining is the cell nucleus. Scar bar = 10 µm. (**B**) Cell proliferation of the NSCs cultured on RH40 and RH70 fibers. TCP stands for tissue culture plate. (**C**) Cell migration of the NSCs cultured on RH40 and RH70 fibers (* *p* < 0.05, ** *p* < 0.01).

One of the challenges in nerve tissue engineering is to produce a scaffold with low bacteria colonization and promote cell behaviors as well. Figure 6A shows the morphology of NSCs attached to RH40 and RH70 nano-pore nanofibers for 1 day. On the aligned fibers, the NSCs were elongated in the fiber direction, indicating that the aligned fibers provided the topographical guidance of NSCs that mimicked the nerve tissues. RH70 nano-pore nanofibers also displayed increased cell adhesion and distribution compared to the RH40 nano-pore nanofibers, which may have been since the former offered a greater surface for cell attachment. Furthermore, Figure 6B shows that two nano-porous fibers were conducive to cell proliferation, and in particular, the RH70 fibers were more predisposed to cell growth. This result is similar with those of previous studies, in which cells responded better to the larger nano-pore fibers surfaces owing to their higher roughness [17,18]. Despite the RH70 fibers reducing bacteria colonization, they did not inhibit the NSC proliferation. It is likely that the dimensions of NSCs could allow a few focal adhesion points on the fibers through filopodia and lamellipodia formation, enabling their attachment and growth [32]. These data demonstrated that large nano-pores on aligned fibers could enhance cell adhesion and proliferation, which was beneficial to ECM depositing and tissue remodeling. However, it was remarkable to note that NSCs preferred to migrate on RH40 rather than RH70 fibers (Figure 6C), indicating that small nano-pores on aligned PLLA fibers were beneficial for cell migration.

**Figure 7:**
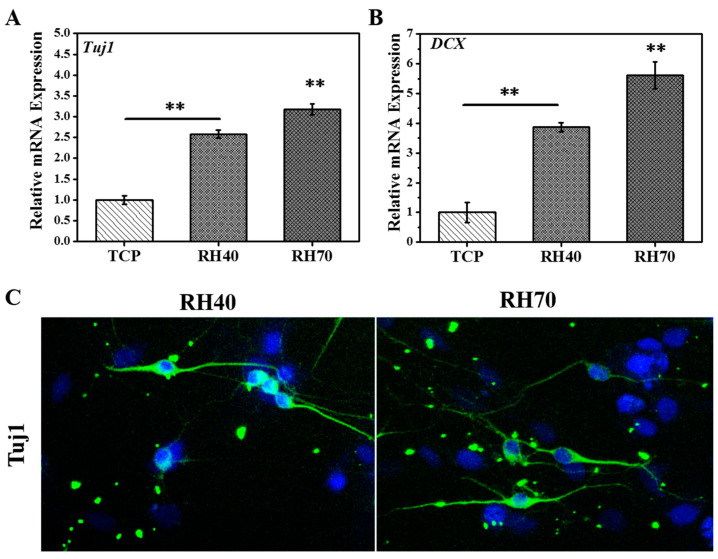
mRNAs of *β-Tubulin* (*Tuj1*) (**A**) and Doublecortin (*DCX*) (**B**) of NSCs expression on different substrates after 7 days of culture. TCP means tissue culture plate. (**C**) Immunocytochemical analysis of the expression of Tuj1 protein in NSCs on RH40 and RH70 fibers after 7 days of culture (** *p* < 0.01).

β-Tubulin (Tuj1) and Doublecortin (DCX) are commonly associated with NSCs and are expressed at the stage of neuron precursor cells [40,41]. RT-PCR was employed to further survey the impact of varied nano-porous fibers on NSCs. Figure 7A,B showed that Tuj1 and DCX in NSCs from larger nano-porous fibers, especially in the case of RH70 fibers, were suggestive of promoting even higher expression in NSCs. Tuj1-representative images of the stained NSCs on different matrices, obtained after a 7-day culture, are presented in Figure 7C. It can be observed that the existence of larger nano-pores on well-aligned fibers remarkably influenced Tuj1 protein expression. It should further be highlighted that an intense expression of the associated protein marker by the NSCs was noted on the RH70 fibrous matrices compared to those of RH40. This may therefore serve as a preliminary indicator of success in employing the larger, nano-porous surface-designed fibers to facilitate NSC differentiation. In the current stage, the mechanisms about the nano-pore RH70 fiber induction effect were not totally clear, and future studies should be performed to investigate some probably involved signal transduction pathways. For example, the existing studies showed that some signal transducers, including Rho proteins/Rho-associated protein kinase (RhoA/ROCK) and phosphatidylinositol 3-kinase/protein kinase B (PI3K/AKT) [18,42], are involved in NSC differentiation when a nanotopography was applied.

## 3. Materials and Methods

### 3.1. Generation of Well-Aligned Nano-Porous PLLA Fibers

Well-aligned nano-porous PLLA (M_η_ = 500 kDa, Jinan Daigang Biomaterials Co., Ltd., Jinan, China) fibers were prepared using a PLLA/dichloromethane (DCM; Merck, Germany) solution via the stable jet electrospinning (SJES) method [18]. Briefly, 0.3 g PLLA was firstly dissolved in 10 mL DCM under stirring at ambient temperature (20–25 °C) for 12 h. A 10 mL syringe with an 18-gauge flat needle was used to load the solution and the feeding rate was set to 1.0 mL/h with a syringe pump (Longer, Baoding, China). The PLLA solution was electrospun into various nano-pore fibers at room temperature (20–25 °C) under the following parameters: high-voltage power supply (13 kv), tip-to-collector distance (24 cm), rotating drum speed (600 rpm), and relative humidity (40% or 70%, termed as RH40 or RH70).

### 3.2. Characterization

To endow the fibers with improved conductivity, we sputter-coated gold onto the fiber surface for 50 s. The fiber morphology was determined using scanning electron microscopy (SEM) at 8–10 kV. Atomic force microscopy (AFM) was used to detect fiber profile surface roughness by NanoScope IV (Veeco, Plainview, NY, USA). The scanning probe microscope used in AFM was Dimension 3100. A tapping mode was then applied to scan the samples in the air. The roughness value (Ra) was calculated as the average value of Z-trace, which was calculated through AFM images.

X-ray diffraction (XRD) spectroscopy was performed on a Rigaku D/max 2550 PC with Cu Ka radiation, operated at 40 kV, 30 mA, and a 5°(2ϑ) per min scanning rate. Differential scanning calorimetry (DSC; Q10, TA instruments, New Castle, DE, USA) was employed to determine the thermal parameters of the prepared fibers, which were as follows: heating rate: 10 °C/min, temperature: from −150 to 250 °C, and N_2_ atmosphere: flow rate: 50 mL per min. The weight of the sample increased from 8 to 10 mg. Utilizing the sessile drop method, the static water contact angle (WCA) of varied nano-porous fibers was measured by video-enabled goniometer (VCA-optima, AST, Pittsburgh, PA, USA). A distilled water drop (0.25 mL) was placed at all 8 locations per sample. The fibers’ shapes did not change noticeably once they had affixed themselves to the fibrous membrane surface. The projected images of the droplets were analyzed to define their contact angles, and crystallinity of each sample was ascertained using the following equation [35]:

*X_c_*% = [(Δ*H_m_*−Δ*H_c_*)/93] × 100% (1)

where Δ*H_m_* represents the fusion heat of the fusion endothermic peak and Δ*H_c_* represents the fusion heat of the raw PLLA of 100% crystalline.

### 3.3. Bacterial Activity and SEM

*Escherichia coli* (*E. coli*, ATCC 8739) or *Staphylococcus aureus* (*S. aureus*, CMCC 26003) (10^4^ cells/mL) were cultured for 14 h in 3% tryptic soy broth (TSB) medium. The RH40 and RH70 fibers were then placed into culture plates with 24 wells, to which 1000 μL bacterial solution was added. After 24-h culture, all bacteria were removed from the samples with gentle washing with phosphate-buffered saline (PBS). The solution suspension was diluted serially (10×, 100×, 1000×) and subsequently laid on a tryptic soy agar plate in 10 μL aliquots, and cultured for 14 h at 37 degrees Celsius. The colony-forming units (CFUs) were calculated. A PBS solution including 2.5% glutaraldehyde and 4% paraformaldehyde was used to fix the bacteria for 1 h. Then, acetone solutions were applied to dehydrate the above bacteria solution until 100% was achieved, followed by rinsing with acetone with hexamethyldisilane (HMDS) (1:1) and pure HMDS for 10 min. Lastly, SEM was employed to observe bacterial morphology.

### 3.4. Cell Culture and Seeding

Mouse NSCs (MUBNF-01001, cyagen, China) were preserved in Eagle’s minimum essential medium (EMEM; ATCC) containing 10% fetal bovine serum (FBS, Zhejiang Tianhang Biotechnology Co., Ltd., China) as well as 1% penicillin/streptomycin (Tianjin Haoyang Biological Products Technology Co., Ltd., China) at 37 °C, and with a CO_2_ concentration of 5%. Prior to cell seeding, nanofibrous scaffolds underwent sterilization with 30-min ultraviolet (UV) irradiation, 30-min 70% concentration ethanol, 15-min rinse, and immersion in PBS. Afterwards, NSCs (1 × 10^4^ cells) were seeded onto the nanofibrous scaffolds in a 500 µL medium of a 24-well plate. By using the cell counting kit-8 (CCK-8, Dojindo, Japan), we then assayed the proliferation of cells at 1, 4, and 7 days.

### 3.5. Cell Morphology

Cell morphology was analyzed after 1 day. To sum up, 4% (w/v) paraformaldehyde was added for the 30-min fixation of the cells, which were then rinsed 3 times using PBS. 0.1% (v/v) Triton^X-100^ (Aldrich, St. Louis, MO, USA) was subsequently utilized to permeabilize the cells for 15 min, followed by a triple wash with PBS. Thereafter, the actin was stained with fluorescein isothiocyanate (FITC)-conjugated phalloidin (diluted in PBS at 1:40, Gibco, Waltham, MA, USA) for 30 min in the dark environment, after which the nucleus was stained with 4,6-diamidino-2-phenylindole (DAPI; concentration: 0.8 mg/mL) (Gibco, Waltham, MA, USA) for 15 min.

### 3.6. Cell Migration

To assay cell migration, we produced cell-free wound-like gaps by customized steel loops with a (1 mm) central barrier. In summary, NSCs (2 × 10^5^ cells) were seeded onto each nanofibrous scaffold using the steel loops, and distributed over plates comprising 24 wells, before being cultured in the standard medium for about 3 h. This serum-free medium was subsequently used for a further 36-h culture to suppress cell proliferation. The steel rings were then removed and cells were subjected to 36-h culture. Lastly, DAPI staining was conducted to observe migration.

### 3.7. Reverse Transcription Polymerase Chain Reaction (RT-PCR)

On the basis of the manufacturer’s protocol, we employed the RNA extraction kit (Takara, Japan) to extract the total RNA from NSCs, and the High Capacity Complementary DNA (cDNA) Reverse Transcription kit (AB Applied Biosystems, Carlsbad, CA, USA) was utilized for reverse-transcribing the first strand cDNA. The ABI Prism 7500 Sequence Detection System with SYBR Green Supermix was used to conduct RT-PCR, the primers of which are tabulated in Table 1. In addition, the 2^−^^ΔΔCt^ method was applied to evaluate the relative mRNA expression levels of a target gene, and the housekeeping gene GAPDH was taken as an internal reference.

### 3.8. Immunocytochemistry

Once NSCs had been cultured for 7 days, a β-Tubulin (Tuj-1) neuronal marker was used for immunostaining. We utilized 4% paraformaldehyde for the 30-min fixation of the cell scaffold, which was later permeabilized by 0.5% Triton^X-100^ for about 15 min. NSCs were thereafter blocked with 1% bovine serum albumin (BSA) for 30 min. Subsequently, a primary antibody (Tuj-1, Abcam, United Kingdom) was used to stain the samples for 1 h, which were then dyed with a secondary antibody (Rabbit Anti-Mouse IgG (H+L)-FITC, USA) for the same duration. Furthermore, the nuclei were stained with 4′,6-diamidino-2-phenylindole (DAPI) prior to examination through a confocal microscope (Nikon, Japan).

### 3.9. Statistical Analysis

Quantitative data were all expressed as means ± standard deviation. To determine differences between groups, we used Origin 8.0 software to carry out the statistical analysis with ANOVA of Tukey’s test. When *p* < 0.05, it was considered statistically significant.

## 4. Conclusions

In summary, the novel SJES method was employed to easily produce well-aligned PLLA fibers with uniform ellipsoidal-shaped nano-porous surface textures, wherein the nano-pore sizes were determined by variable ambient humidity conditions during the SJES process. The greater the ambient humidity, the larger the nano-pore sizes. The roughness of fiber surfaces was also enhanced by the increase in ellipsoidal nano-pores. For example, the size and surface roughness produced on the RH40 fibers were 1.4 ± 0.3 μm and 66 ± 10 nm in an ambient humidity of 40% during the SJES process, respectively. For RH70, they were 1.7 ± 0.2 μm and 122 ± 27 nm at an ambient humidity of 70%, respectively. These data results were noteworthy in that RH70 fibers decreased bacterial colonization for *S. aureus* compared to RH40 fibers, but neither of these two fibers had any inhabit bacterial effect on *E. coli*. Moreover, the ellipsoid nano-porous fibers impacted cellular responses including cell adhesion, migration, proliferation, and differentiation, as well as the expression of desired NSC phenotype markers. Compared to the small nano-pores, the large ones proved more beneficial in terms of the adhesion, proliferation, and differentiation of NSCs. However, NSCs preferred migrating on the aligned fibers with a small ellipsoid nano-porous surface texture. The authors contend that together with a uniform nano-porous surface texture, well-aligned electrospun fibers could serve as a promising biomaterial with NSC responses and low bacterial colonization. The proposed method could be applied to achieve improved and adjustable cellular responses in nerve tissue engineering.

## Figures and Tables

**Table 1:** Primer sequences of specific genes for quantitative RT-PCR analysis.

Genes	Forward Primer Sequence (5’–3’)	Reverse Primer Sequence (5’–3’)
*Tuj 1*	ACTTTATCTTCGGTCAGAGTG	CTCACGACATCCAGGACTGA
*DCX*	CAGAAGCCATCAAACTGGA	AATCATGGAGACAAGTTACCTG
*GAPDH*	TGACCTCAACTACATGGTCTACA	CTTCCCATTCTCGGCCTTG

## Data Availability

All relevant data is presented in the manuscript, raw data is available upon request from the corresponding author.
